# Not Everybody Sees the Ness in the Darkness: Individual Differences in Masked Suffix Priming

**DOI:** 10.3389/fpsyg.2016.01585

**Published:** 2016-10-14

**Authors:** Joyse Medeiros, Jon Andoni Duñabeitia

**Affiliations:** Basque Center on Cognition, Brain and LanguageDonostia, Spain

**Keywords:** morphological processing, individual differences, suffix priming, masked priming, semantics

## Abstract

The present study explores the role of individual differences in polymorphemic word recognition. Participants completed a masked priming lexical decision experiment on suffixed words in which targets could be preceded by suffix-related words (words sharing the same suffix) or by affixed primes with a different suffix. Participants also completed a monomorphemic word lexical decision and were divided in two groups (fast and slow readers) according to their performance in this task. When the suffix priming data were analyzed taking into consideration participants' reading speed as a proxy for their greater reliance on orthography or on semantics, a significant interaction between reading speed and the magnitude of the masked suffix priming effects emerged. Only slow participants showed significant priming effects, whereas faster participants showed negligible masked suffix priming effects. These results demonstrate that different reading profiles modulate the access to morphological information in a qualitatively different manner and that individual differences in reading determine the manner in which polymorphemic words are processed.

## Introduction

Since the seminal study by Taft and Forster (1975), many studies in different languages have supported the view of a morphological decomposition process mediating lexical access for polymorphemic words (see Rastle and Davis, 2008; Amenta and Crepaldi, 2012, for reviews), and together with sub-lexical and lexico-semantic variables, the morphological richness of words is a key factor in visual word recognition (e.g., Baayen et al., 2006). The evidence gathered from numerous masked priming studies has reinforced the assumption of automatic decomposition of morphologically complex words (e.g., Grainger et al., 1991; Taft, 2003; Rastle et al., 2004; Taft and Kougious, 2004; Longtin and Meunier, 2005). It is now well known that prime-target pairs sharing their root morpheme (e.g., *walker-WALK* or *revive-SURVIVE*) activate each other, demonstrating that affixed words are decomposed into their corresponding morphemes (e.g., *walk*+*er*; e.g., Rastle et al., 2000; Longtin et al., 2003; Pastizzo and Feldman, 2004). Similarly, polymorphemic words sharing derivational suffixes (e.g., *walker*-*DREAMER*) also activate each other, yielding masked suffix priming effects that emerge from the automatic decomposition of polymorphemic words (e.g., Duñabeitia et al., 2008). Finally, compound words sharing one of their constituent lexemes (e.g., *milkman*-*FIREMAN*) have been shown to activate each other, demonstrating that morphemic parsing extends to other forms of polymorphemic words too (e.g., Duñabeitia et al., 2009; Crepaldi et al., 2013).

In contrast to purely post-lexical decompositional views of polymorphemic words (e.g., Marslen-Wilson et al., 1994; Rueckl and Raveh, 1999; Giraudo and Grainger, 2000; Plaut and Gonnerman, 2000; Davis et al., 2003), the largest body of evidence gathered in the last few years demonstrates that polymorphemic words are accessed through their constituent morphemes. Yet, some authors posit that both early and late decomposition mechanisms may guide the recognition of polymorphemic words (e.g., Baayen et al., 1997; Diependaele et al., 2009). Proponents of this view defend an early semantically blind decomposition process operating mainly based on morpho-orthographic information, but also assume a morpho-semantic stage in which semantic information plays a role in polymorphemic word processing. Along this line, the Diependaele et al. (2009) hybrid model proposes that during lexical access morphological information is mapped in parallel into morpho-orthographic and morpho-semantic routes. The first route operates at the level of sub-lexical orthographic representations, and therefore, it is semantically blind. The second mechanism involves the processing of regularities in the mapping of word forms onto semantics, thus being sensitive to whole-word effects and to top-down processes. By assuming the existence of these two processing stages, one could account for decomposition effects in pseudo-complex words (e.g., *corner* primes *CORN* in spite of the lack of semantic relationship between these two lexical items) as well as for transparency effects (e.g., the priming effect between *walker* and *WALK* is larger than the priming effect between *corner* and *CORN*). In this line, the priming effects for morphologically opaque relationships may result from morpho-orthographic computation, while the larger effects found for transparent morphological relationships may result from the enhanced morpho-semantic information they provide.

Critically, recent studies have demonstrated that individual differences across readers result in different degrees of reliance on morpho-semantic and morpho-orthographic pieces of information, depending on the reading strategy followed by each person. Stemming from the seminal unmasked semantic priming results reported by Yap et al. (2009), and from the masked form priming effects reported by Andrews and Hersch (2010), Andrews and Lo (2013) conducted a masked priming lexical decision experiment aimed at disentangling the underlying factors that could have led to partially contradictory morphological priming effects previously reported in the literature. In recent years, the evidence on morphological priming between morphologically complex affixed words and their stems (e.g., *walker-WALK*) and between pseudo-derived words and their pseudo-stems (e.g., *corner-CORN*) has offered inconsistent results, with some studies reporting effects of similar magnitude (e.g., Devlin et al., 2004; Rastle et al., 2004) and other studies reporting larger effects for truly derived items than for pseudo-derived items (e.g., Feldman et al., 2009; Diependaele et al., 2011). By comparing transparent (*teacher-TEACH*), opaque (*coaster-COAST*) and form primes (*pulpit-PULP*), Andrews and Lo found stronger priming effects for transparent than for opaque and form-related pairs in their general analysis on the results averaged across all participants, regardless of their reading ability. More importantly, when participants' performance on vocabulary and spelling tests was further considered, the authors demonstrated that readers with a semantic profile (i.e., individuals with better vocabulary than spelling skills) showed larger priming effects for transparent as compared to opaque and form-related primes (namely, a transparency effect). In contrast, participants with an orthographic profile (i.e., individuals with better spelling than vocabulary skills) showed similar priming effects for opaque pairs and transparent pairs.

Similarly, a recent study by Duñabeitia et al. (2014) explored whether individual differences in reading strategies could be responsible for some inconsistent results previously found in the literature on morphological decomposition: the difference between transposed-letter priming effects across and within morphemes. Duñabeitia et al. (2007) replicated previous findings of transposed letter (TL) priming effects for polymorphemic words (*vio****il****nist-VIOLINIST*; see Christianson et al., 2005), and showed that the priming effect disappeared when the transposition was inserted between two morphemes (e.g., *violi****in****st-VIOLINIST* vs. *violi****er****st-VIOLINIST*). In contrast, Sánchez-Gutiérrez and Rastle (2013) did not find any difference in the magnitude of the TL effects when transposing letters within and between morphemes in a masked priming experiment, in line with other similar studies (e.g., Rueckl and Rimzhim, 2011; Beyersmann et al., 2012; Masserang and Pollatsek, 2012; Beyersmann et al., 2013). Following Andrews and Lo (2013), Duñabeitia et al. (2014) decided to investigate whether individual differences in orthographic processing could be responsible for this apparent inconsistency. They designed a masked transposed-letter priming lexical decision experiment with 420 suffixed Spanish words and tested 80 participants who were further divided in two groups following a median-split procedure based on their speed of response in the task. Results showed that while slower readers did not show differences in the magnitude of transposition priming effects either between or within morphemic boundaries, faster readers presented larger priming effects for transpositions occurring within than between-morphemes. Duñabeitia et al. (2014) thus concluded that TL effects across morphemic boundaries might be better depicted as a continuum of individual differences in participants' reading profiles, and especially, in their reliance on morpho-orthographic information.

Hence, as seen, recent evidence suggests that morphological decomposition processes may depend on individual reading profiles (i.e., the greater or smaller reliance on semantic vs. orthographic information across readers), corroborating the idea that such individual differences in reading must be incorporated in the models that aim to explain the putative role of orthographic and morphological constraints in polymorphemic word recognition. The aim of the present study was to investigate the role of individual differences in masked suffix priming effects.

Chateau et al. (2002) found masked morphological priming effects with words sharing the initial prefixes (e.g., *dislike-DISPROVE*), but not for those with initial orthographic overlap (e.g., *element-ELEVATOR*; see also Giraudo and Grainger, 2003, to some extent). Marslen-Wilson et al. (1996) also found a significant priming effect for pairs sharing suffixes (e.g., darkness-toughness) in a cross-modal priming experiment. In a series of masked priming lexical decision experiments, Duñabeitia et al. (2008) demonstrated that prime-target word pairs that shared their suffix (e.g., *darkness-HAPPINESS*) yield significant priming effects as compared to word pairs sharing only orthographic overlap. Whereas, there seems to exist certain limits to masked suffix priming effects in specific languages (e.g., see Giraudo and Grainger, 2003, for an illustrative example of this issue in French), these effects have been found to be relatively robust in other languages (e.g., Spanish: Duñabeitia et al., 2008; English: Crepaldi et al., 2016). Considering that masked suffix priming effects significantly differ in magnitude from those obtained between orthographically overlapping strings (e.g., the non-word “*sportel*” does not prime the monomorphemic word *BROTHEL*, while the seemingly polymorphemic non-word “*sheeter”* primes *TEACHER*; see Crepaldi et al., 2016), Duñabeitia et al. suggested that these masked suffix priming effects are exclusively mediated by morpho-semantic processes. However, it should be acknowledged that the extent to which these effects are morphological in essence, or alternatively, semantically driven (parallel to the relationship of compound word pairs like *milkman* and *postman*; see Duñabeitia et al., 2009) is still controversial. Crepaldi et al. demonstrated that masked suffix priming effects are position-specific, since affixes at (non-)word initial positions did not facilitate the processing of polymorphemic words with that same affix at word-final position (e.g., *ersheet-TEACHER*). Nonetheless, be they morphological or semantic in essence, the critical piece of information is that these effects are not orthographically driven.

In the current study we explored the role of individual differences in reading for the emergence of masked suffix priming effects. Suffixed Spanish words that could share their suffixes were used as primes and targets, and a group of 130 native Spanish speakers were tested. Considering that word pairs that share their orthographic endings and pseudo-suffixed word pairs that share their endings do not yield significant priming effects (see Marslen-Wilson et al., 1996; Reid and Marslen-Wilson, 2000; Duñabeitia et al., 2008; Crepaldi et al., 2016), while target words preceded by (non-)words that share the suffix do, it seems reasonable to assume that the locus of the suffix priming effect is morphological (or morpho-semantic) in essence. In order to divide these participants according to their reading profiles and to characterize them according to their potential reliance on morphological (or morpho-semantic) information, they also completed a lexical decision task that exclusively included monomorphemic words.

Recent research has established a close relationship between reading speed and the reliance on orthographic representations, so that a better performance in tasks measuring orthographic processing typically predicts shorter overall reading times and better reading fluency (see Saiegh-Haddad, 2005, for a study demonstrating a correlation of *r* = 0.75 between letter recoding, conceived as an orthographic task, and the number of words that children could read accurately per minute; see also Wimmer et al., 2000; Müller and Brady, 2001). Furthermore, reading speed in the lexical decision task varies as a function of orthographic skills, as demonstrated by the study exploring Scrabble players' performance in an adapted version of this task conducted by Hargreaves et al. (2012). Hargreaves and colleagues showed that readers with increased lexical knowledge and enhanced orthographic skills (namely, expert Scrabble players) presented faster reading times than control readers. More importantly for the purposes of the current study, they also demonstrated that the faster readers were the ones showing the smallest semantic effects. Reduced concreteness effects were found for expert Scrabble players than for non-expert controls, reinforcing the view that the augmented capacity to encode orthographic information shown by these readers reduces the reliance on the meaning of words (i.e., the so-called “*semantic deemphasis*”; see also Novick and Sherman, 2008). This effect is in line with preceding research suggesting that the magnitude of semantic effects varies as a function of reading speed (e.g., Rodd, 2004; see also Yap et al., 2016). Hence, it can be established that enhanced orthographic skills result in shorter reading times, which in turn yield reduced semantic effects. Following this same rationale and extending these hypotheses to the field of morphological processing, a greater reliance on morpho-orthographic information has been suggested for faster readers, while a greater reliance on morpho-semantic information has been suggested for slower readers (see Duñabeitia et al., 2014).

Hence, in light of existing evidence suggesting (1) that the magnitude of semantic effects are inversely associated with reading speed (cf. Hargreaves et al., 2012), and (2) that suffix priming mainly relies on semantically overlapping morphological representations (cf. Duñabeitia et al., 2008; see also Crepaldi et al., 2016), we expected a modulation of participants' suffix priming effects based on their reading speed. We predicted that the reading profile of the participants (mainly orthographic vs. mainly morpho-semantic; fast vs. slow) would influence the magnitude of the priming effects elicited by the pairs sharing the same suffix. We hypothesized that readers primarily focusing on morphological information (namely, slow readers) would show greater masked suffix priming effects than readers with a more marked (morpho-) orthographic profile (namely, fast readers).

## Methods

### Participants

130 native Spanish speakers (81 females) with a mean age of 22.85 years (*SD* = 3.42) completed this experiment. All of them had normal or corrected-to-normal vision and signed informed consent forms prior to the experiment.

### Materials

For the masked suffix priming lexical decision experiment a set of 500 Spanish suffixed words (250 primes and 250 targets) were selected. The set of words included 23 different Spanish suffixes (ez, ario, ato, azo, dad, dero, dor, dura, eño, ería, ero, ez, iego, ismo, ista, itis, mento, nte, ón, oso, torio, udo, ura; see Appendix in Supplementary Material), and the suffix length ranged from 2 to 5 letters (mean = 3.2; *SD* = 0.6). The characteristics of the items used as primes and targets are presented in Table 1. Two experimental lists were created following a counterbalanced design. Word prime-target pairs were created by arranging suffix-related items together (50% of the word pairs, yielding 125 related prime-target pairs in each list; e.g., *herrero-BASURERO*), or by mixing item pairs with different morphological endings (50% of the word pairs, yielding 125 unrelated prime-target pairs). As expected, the orthographic overlap between related and unrelated pairs significantly differed, reflecting a greater overlap between pairs that shared their suffix as compared with pairs not sharing the suffix. An analysis of the Levenshtein distance (the number of edits needed in one string to end with the other) showed that unrelated pairs required on average 8.14 edits (*SD* = 1.41), while related pairs only required 4.78 edits (*SD* = 1.25), which was significantly different [*t*_(249)_ = 33.13, *p* < 0.001]. The list of primes and targets did not differ in any other of the factors mentioned above (all *p*s > 0.95 and *t*s < 1). Hence, half of the words shared the same suffix with their primes, while the other half of the words was preceded by strings with an unrelated ending (following a counterbalanced Latin square design). Additionally, 500 pseudowords matched in length and syllabic structure to the words were created using *Wuggy* (Keuleers and Brysbaert, 2010). Pseudowords were arranged following the same criteria used with the words (e.g., unrelated pseudoword pairs: *bematero-POFINADOR*; related pseudoword pairs: *butenlez-SOGOSTEZ*). The final list of items contained 250 word targets and 250 pseudoword targets.

**Table 1:** **Characteristics of the words used in the suffix priming lexical decision task (primes and targets), and in the monomorphemic lexical decision task**.

	**Suffix Priming LDT**		**Monomorphemic LDT**
	**Primes**	**Targets**	
Frequency (per million)	4.16 (7.19)	4.17 (6.20)	8.95 (3.17)
Length (number of letters)	8.74 (1.45)	8.74 (1.45)	5.00 (0.00)
Neighbors (Coltheart's N)	0.48 (0.91)	0.56 (0.81)	3.50 (3.28)

*Standard deviations are shown within parenthesis. Values were obtained from Davis and Perea (2005)*.

For the monomorphemic word lexical decision test, 50 5-letter Spanish words were selected (see Table 1 for the characteristics). These fifty words were used to create fifty pseudowords in *Wuggy* (Keuleers and Brysbaert, 2010), leading to the final set of pseudowords matched in length and bigram frequency to the words.

### Procedure

The whole experimental session was held in individual soundproof test cabins, on Dell® Optiplex 760 PCs with CRT monitors (1024 × 768 resolution with a refresh rate of 100 Hz) with DMDX software (Forster and Forster, 2003). In both lexical decision tasks, participants saw strings of letters in the center of the screen and they had to indicate if they were real Spanish words or not by pressing a green button in a response box for real words and a red button for pseudowords. They were instructed to respond to the target strings as fast and as accurately as possible. Participants first completed the masked suffix priming lexical decision task. Each trial started with the presentation of a mask (#######) for 500 ms, immediately followed by the prime in lowercase Courier New that was displayed for 50 ms (5 cycles). Then, the target appeared in uppercase letters and stayed on the screen for a maximum of 2000 ms or until a response was given. The length of the mask varied from trial to trial depending on the number of characters of the primes and targets. After completing this task, participants completed the short lexical decision task including monomorphemic words and pseudowords. The strings were presented in the center of the screen after an initial fixation mark (“+”) that was presented for 500 ms. Items were presented in uppercase Courier New for a maximum of 2000 ms or until a response was given. Every task started with four practice trials. The items were presented in a random order and the whole session lasted approximately 20 min.

## Results

Latency analysis excluded erroneous responses (4.24%) as well as RTs that did not fall within the mean plus/minus 2.5 *SD*s range obtained for each participant in each condition (2.89% of the data). Mean RTs and error rates are presented in Table 2. Two different sets of analyses were carried out on the resulting trimmed data. First, an ANOVA approach was followed, categorizing the participants as a function of their reading speed. To this end, the 130 participants were categorized as fast or slow as a function of their mean RT in the monomorphemic lexical decision task. In order to do so, a median-split procedure was followed (see Häikiö et al., 2009; Duñabeitia et al., 2014). And second, we followed an approach based on generalized linear mixed-effect models (GLMM), using participants' mean RTs in the monomorphemic lexical decision task as a continuous fixed factor. As discussed by MacCallum et al. (2002), the admittedly artificial dichotomization of a quantitative variable that is continuous in essence (namely, speed of response) could yield negative statistical consequences. Hence, we took a closer look at how the suffix priming data were modulated as a function of the non-dichotomized measure of reading speed. Instead of using linear mixed-effect models (LMM), we opted for GLMM given that they are better suited for investigating individual differences by satisfying normality assumptions without requiring data transformation (see Lo and Andrews, 2015, for further discussion).

**Table 2:** **Average reaction times (in milliseconds) and error rates (percentage) for each reader type and condition in the suffix priming experiment**.

		**Related**	**Unrelated**	**Priming**	**Non-words**
All	RTs	708 (125)	715 (130)	7	910 (250)
	%Error	3.66 (2.89)	3.88 (2.98)		4.72 (5.71)
Faster	RTs	625 (89)	626 (90)	1	735 (140)
	%Error	3.93 (3.00)	4.21 (3.10)		3.89 (4.80)
Slower	RTs	792 (97)	803 (101)	11	1085 (209)
	%Error	3.38 (2.78)	3.54 (2.85)		5.55 (6.40)

*Standard deviations are shown within parenthesis. The priming effect was calculated by subtracting the Related condition from the Unrelated condition*.

After excluding latencies associated with erroneous or outlier responses, each participant was assigned to a particular group (faster or slower reader) according to their mean reaction time (RT) in the monomorphemic lexical decision task (dichotomized variable). Participants with mean RTs higher than the median RT (659 ms; mean = 684 ms, *SD* = 137 ms) were assigned to the slower group, and participants with mean RTs lower than the median RT for the whole group were assigned to the faster group. The slower group (*N* = 65) had a mean RT of 784 ms (*SD* = 120 ms) and the faster group (*N* = 65) had an average RT of 581 ms (*SD* = 47 ms). ANOVAs were then run on the word data from the masked suffix priming lexical decision task following a 2^*^2^*^2 design, including the factors Relatedness (Related|Unrelated), Reader Type (Faster|Slower), and List (1|2) (see Table 2). ANOVAs on the RTs revealed a main effect of Relatedness [*F*1_(1, 126)_ = 10.47, *p* = 0.002, $\mu_{\text{partial}}^{2}=$ 0.077, 1−β = 0.895; *F*2_(1, 248)_ = 8.82, *p* = 0.003, $\mu_{\text{partial}}^{2}=$ 0.034, 1−β = 0.841], demonstrating that words preceded by suffix-related masked primes were recognized significantly faster than words preceded by morphologically unrelated primes (an overall 7 ms difference). Not surprisingly, a main effect of Reader Type was also found [*F*1_(1, 126)_ = 110.615, *p* < 0.001, $\mu_{\text{partial}}^{2}=$ 0.467, 1−β = 1; *F*2_(1, 248)_ = 3686.17, *p* < 0.001, $\mu_{\text{partial}}^{2}=$ 0.937, 1−β = 1], confirming that the mean response latencies of the readers in the fast group were shorter than those of the readers in the slow group. Importantly, the interaction between Relatedness and Reader Type resulted significant [*F*1_(1, 126)_ = 6.39, *p* = 0.013, $\mu_{\text{partial}}^{2}=$ 0.048, 1−β = 0.708; *F*2_(1, 248)_ = 6.27, *p* = 0.013, $\mu_{\text{partial}}^{2}=$ 0.025, 1−β = 0.703]. Separate analyses were conducted to elucidate the source of this interaction. Slower readers presented a significant main effect of Relatedness [*F*1_(1, 63)_ = 14.22, *p* < 0.001, $\mu_{\text{partial}}^{2}=$ 0.184, 1−β = 0.960; *F*2_(1, 248)_ = 10.84, *p* = 0.001, $\mu_{\text{partial}}^{2}=$ 0.042, 1−β = 0.907], showing a significant masked suffix priming effect (11 ms). In contrast, faster readers did not show any reliable effect of Relatedness (a negligible 1 ms difference) [*F*1_(1, 63)_ = 0.30, *p* = 0.585, $\mu_{\text{partial}}^{2}=$ 0.005, 1−β = 0.084; *F*2_(1, 248)_ = 0.834, *p* = 0.362, $\mu_{\text{partial}}^{2}=$ 0.003, 1−β = 0.149]. ANOVAs on the error rates did not show any reliable effect.

**Figure 1:**
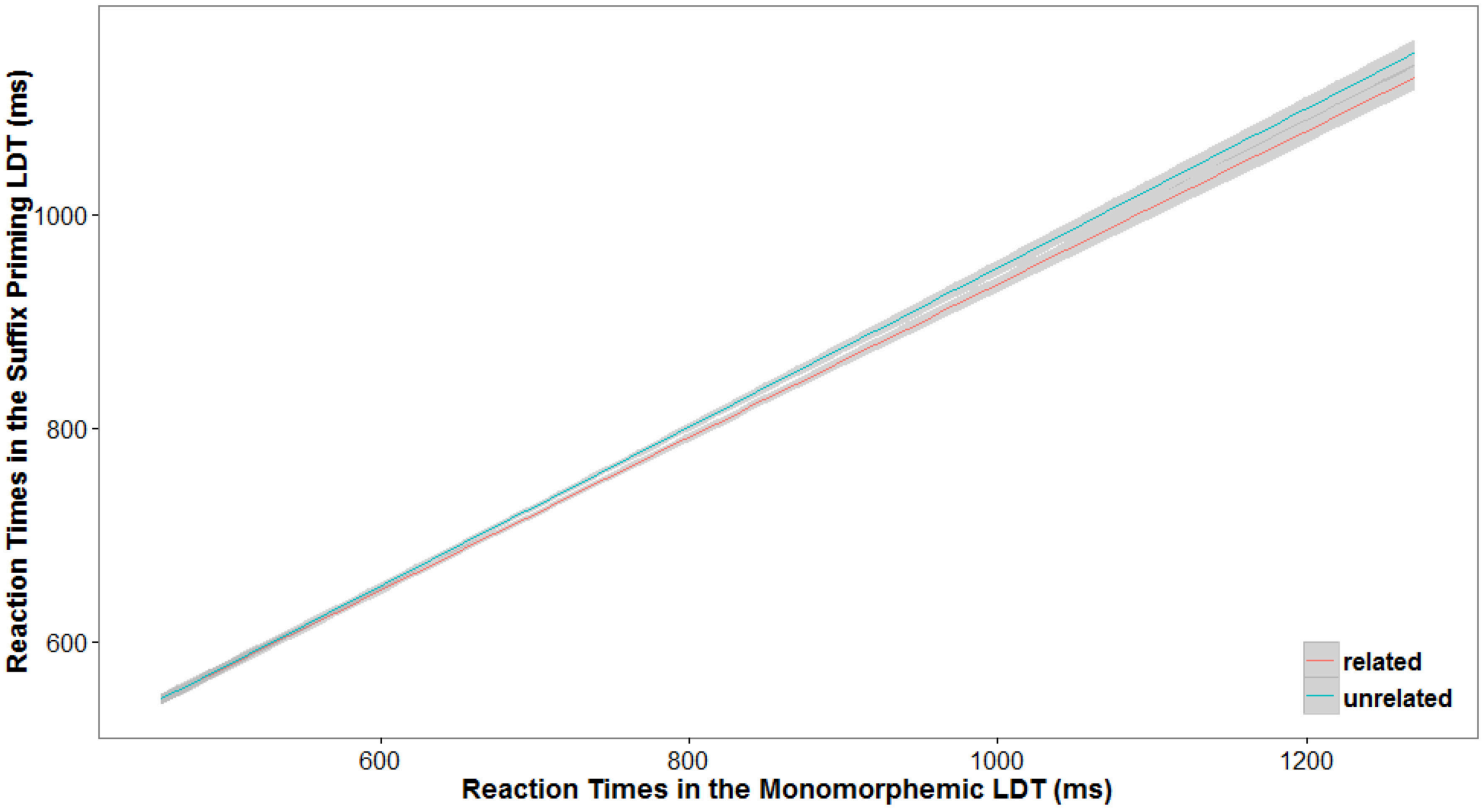
**Results of the GLMM on the latency data in the suffix priming lexical decision task as a function of participants' speed of response in the monomorphemic lexical decision task for the Related and Unrelated prime-target word pairs**. The estimation of the smoothing was done by fitting a generalized additive model.

When the same data were analyzed using GLMM and including participants' mean RTs in the monomorphemic lexical decision task as a quantitative continuous non-dichotomized variable, the same results were found. The analysis was conducted using the *R* program for statistical computing (R Core Team, 2015) and the *lme4* package (Bates et al., 2015). The model used to explain the untransformed RTs in the suffix priming lexical decision task included Relatedness as a fixed factor (Related|Unrelated; with Related as the reference level) together with the mean RT in the monomorphemic lexical decision task (factor Speed), and Items and Participants were added as random factors (see Table 3). An inverse Gaussian distribution of RTs and a linear relationship between the predictors and those RTs (identity link function) were assumed (see Lo and Andrews, 2015). Different model structures were considered, and the data were originally modeled by adding the maximal random slope structure (cf. Barr et al., 2013). However, the inclusion of random slopes for each fixed factor and their interactions resulted in a failure to converge as a consequence of the complexity of the model (for discussion on this issue, see Bates et al., submitted; see also Janssens et al., 2016). Hence, given the convergence problems, a parsimonious simple random-intercept model was created, expressed as Reaction Time ~Relatedness + Speed + Relatedness:Speed + (1 | Participants) + (1 | Items) following the notation used by Bates et al. (2015). As shown in Table 3 and Figure 1, the results were fully congruent with those obtained in the ANOVAs, confirming the modulation of the suffix priming effect as a function of participants' speed of response.

**Table 3:** **Model output for the fixed and random factors**.

**Fixed effects**	**Estimate**	**Standard error**	***t*-value**	**Pr(>|z|)**
Intercept	426.57	5.22	81.67	<0.001
Relatedness	−10.84	5.15	−2.11	0.035
Speed	0.51	0.02	28.60	<0.001
Relatedness^*^Speed	0.02	0.01	2.69	0.007
**Random effects**	**Variance**	**Standard deviation**		
Items	1191	34.51		
Participants	1044	32.31		
Residual	0.06	0.01		

## Discussion

The main aim of the present study was to evaluate the manner in which individual differences in participant's reader profiles modulate masked suffix priming effects. Spanish polymorphemic targets preceded by primes that shared the same suffix were contrasted with targets preceded by morphologically unrelated primes. Results showed an overall small, yet significant, masked suffix priming effect (see also Duñabeitia et al., 2008; Crepaldi et al., 2016). Participants also completed a lexical decision task with monomorphemic items, and were then divided in two groups according to a median-split procedure on their general performance on the task. Participants' reading profiles were then used to estimate if participants in the slower group showed stronger suffix priming effects than participants in the faster group. In line with the initial hypothesis suggesting that the reading profile of the participants (orthographic vs. morphological or morpho-semantic; fast vs. slow) may influence the magnitude of the priming effects elicited by pairs of derived words sharing the same suffix, we demonstrated that the suffix priming effect was significantly larger for the slower than for the faster group (for which no such priming effect was found).

These results are in line with a large body of evidence that suggests that polymorphemic words are decomposed into their constituent morphemes during early stages of visual word recognition (see Amenta and Crepaldi, 2012, for review). The general results from the masked suffix priming lexical decision experiment revealed significant priming effects for word pairs that shared the same suffix, in line with earlier observations (see Crepaldi et al., 2016, for review). This result corroborates the idea that, similar to the case of constituent priming effects in compound words (i.e., postman primes milkman; e.g., Kehayia et al., 1999; Duñabeitia et al., 2009; Crepaldi et al., 2013), smaller (and bound) morphemes such as suffixes are also able to produce priming effects between polymorphemic words. While there are important differences between constituent priming effects and suffix priming effects (e.g., position-specificity; see Crepaldi et al., 2016), these effects suggest the existence of independent representations of morphemic units in the lexicon (Di Sciullo and Williams, 1987; Aronoff, 1994).

More importantly, these results underscore the importance of individual differences in morphological processing. In their seminal study on the effects of individual differences in visual word recognition of polymorphemic words, Andrews and Lo (2013) demonstrated that participants characterized as orthographic readers did not show differences in the magnitude of the priming effects elicited by transparent and opaque primes. In clear contrast, semantic readers (those with lower orthographic skills) showed larger priming effects for transparent than for opaque items. Recently, Duñabeitia et al. (2014) found that faster participants (therefore, readers with an orthographic profile; see Hargreaves et al., 2012) were sensitive to morpho-orthographic interactions, while this was not the case for slower (presumably more semantics-based) readers. The present study adds to the increasing body of evidence on the role of individual differences in polymorphemic word processing showing that slower participants (allegedly the ones less prone to show clear morpho-orthographic effects) show the largest morpho-semantic priming effects, as assessed by suffix priming.

We acknowledge that the inverse relationship between reading speed and sensitivity to (morpho-) semantic levels of processing is not quite well established yet. Hence, the assumption of slower readers showing the largest masked suffix priming effects because of their increased sensitivity to morphological or semantic units is admittedly tentative. Nonetheless, this assumption it is partially supported by preceding research. As discussed in the Introduction, preceding evidence has successfully demonstrated that reading becomes faster as a direct function of a greater reliance on lexical and sub-lexical (e.g., orthographic) information (see Hargreaves et al., 2012). Following this line of argumentation, a previous study demonstrated that faster readers showed larger morpho-orthographic effects (Duñabeitia et al., 2014). Interestingly, past research has also demonstrated that the magnitude of semantic effects decrease as reading speed increases (cf. Hargreaves et al., 2012; see also Rodd, 2004). Considering that masked suffix priming effects are not due to the mere presence of orthographic overlap and that they seem to depend on the presence of shared morphological units (see Duñabeitia et al., 2008; Crepaldi et al., 2016), participants mainly relying on orthographic information (i.e., faster readers) were expected to show reduced priming effects as compared to slower readers. This is precisely what we found in the current study, in which the failure to obtain significant masked suffix priming effects for the faster readers was evident.

While according to our initial hypothesis slower readers were predicted to show larger masked suffix priming effects than faster readers, the full absence of such effects in the latter group was an admittedly surprising and unexpected finding. One possible (yet tentative) way to interpret this finding is to consider that faster readers are partially blind to the morphological units, at least in experimental scenarios using the masked priming technique, and that the relationship they “perceive” between a pair of polymorphemic words like *darkness* and *happiness* is orthographic in nature (i.e., the sequence of overlapping letters “*ness”*), without processing this shared unit as a suffix. Considering preceding evidence demonstrating that word-final orthographic overlap is not sufficient to elicit masked priming effects (see Duñabeitia et al., 2008; Crepaldi et al., 2016), no masked suffix priming effects would be expected for faster readers. However, we prefer to remain cautious at this regard and we refrain from making a strong claim about the full lack of priming effects for this group. Whether small yet significant or negligible effects are found for faster readers, the critical finding shown in the current study is that slower readers show significantly larger masked suffix priming effects, and that these effects are modulated by overall reading speed.

**Figure 2:**
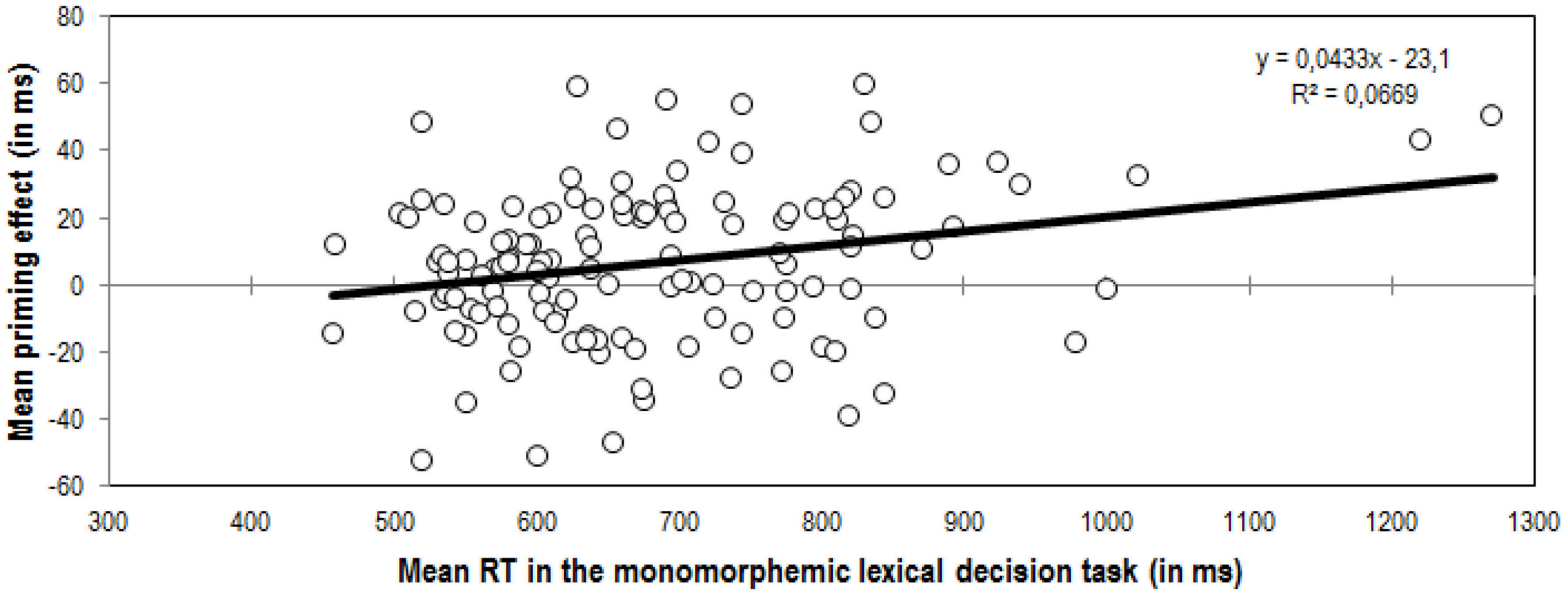
**Correlation between participants' performance in the monomorphemic lexical decision time (mean RT in ms) and their net priming effects in the masked suffix priming experiment (in ms)**. The priming effect was calculated by subtracting the Related condition from the Unrelated condition.

Even though these results are consistent with the predictions and with preceding studies exploring the role of individual differences in morphological processing, a cautionary note on the general relationship between the magnitude of priming effects and participants' speed of response is needed. In the current study, larger masked morphological priming effects were found for the slower participants. In this regard, it is worth noting that the correlation between reading speed (mean RTs) in the monomorphemic lexical decision task and the magnitude of the masked suffix priming effects was significant, yet admittedly modest (*r* = 0.26 *p* = 0.003; see Figure 2). One may wonder whether or not this seemingly direct relationship between general RTs and masked suffix priming effects effectively reflects the reliance of readers with a more semantic profile on morphological units. It has been previously shown that RTs are faster for participants with good spelling skills and good vocabulary (e.g., Yap et al., 2012). In the same line, it has been suggested that good and fast readers show smaller masked priming effects (see Adelman et al., 2014). Hence, as an alternative explanation, one could tentatively argue that the effects reported in the current study are merely the consequence of an inherent direct relationship between general response latencies and masked priming effects, alien to the type of process being explored (i.e., slower subjects show larger effects due to scaling). However, we believe that there are enough reasons to rule out this possibility. As recently demonstrated by Tan and Yap (2016), masked priming effects are not necessarily smaller for highly-skilled readers. Quite on the contrary, Tan and Yap demonstrated that the magnitude of masked repetition priming effects was positively associated with spelling ability and vocabulary knowledge. Besides, as shown in the study by Duñabeitia et al. (2014) exploring the role of individual differences in morpho-orthographic processing, greater masked transposed-letter priming effects are found for transpositions that cross the morphemic boundaries (i.e., transpositions between morphemes) in faster than in slower readers. Hence, the assumption that longer reaction times or impoverished reading fluency yield greater masked priming effects irrespectively of the type of process being explored seems untenable, and we are confident that our results truly reflect the greater reliance on morpho-semantic representations of slower readers.

Altogether, the results of recent studies on the influence of individual differences in polymorphemic word decomposition support the existence of two clearly different processing stages previously described in the literature on morphological processing (see Diependaele et al., 2009): the morpho-orthographic and the morpho-semantic routes. Purportedly, the different effects observed in the literature on polymorphemic word processing seems to depend on the information computed at each of these two stages (see Duñabeitia et al., 2013, for a test of the differential influence of orthographic and semantic processes in accessing morphological information; see Amenta et al., 2015, for a review). On the one hand, morphological priming effects produced by semantically opaque or pseudo-morphological relationships (e.g., corner-CORN) are said to be a by-product of the computations taking place at early morpho-orthographic stages (see Rastle et al., 2004; Andrews and Lo, 2013), as it is also the case for the vanishing of between-morphemes transposed-letter priming effects (e.g., violiinst-VIOLINIST; Duñabeitia et al., 2007, 2014), which has been shown to depend on the degree of reliance on morpho-orthographic information. On the other hand, the processes being primarily computed at morpho-semantic stages of visual polymorphemic word recognition have been claimed to be relatively independent of orthography, such as those elicited by transparent prime-target pairs (e.g., walker-WALK; see Andrews and Lo, 2013), and those elicited by suffix-related prime-target pairs (e.g., darkness-HAPPINESS; see Duñabeitia et al., 2008; Crepaldi et al., 2016). The current study demonstrates that a stronger reliance on each of these different stages of processing (morpho-orthographic vs. morpho-semantic) critically depends on the individual differences in reading speed.

In summary, this study reveals that individual differences in reading profiles (at least, as assessed by reading speed) significantly modulate masked suffix priming effects. Participants with a more marked orthographic profile (or, alternatively, participants with a less clear reliance on morphological information) show negligible masked suffix priming effects. Hence, these results (i) present supportive evidence for the differential role or weight of morpho-orthographic and morpho-semantic information in polymorphemic word processing, and (ii) underscore the importance of assuming (at least) some degree of plasticity in morphological processing, by providing a better characterization of individuals' reading styles.

## Author contributions

JD and JM designed the experiment and prepared the materials. JM collected the data under the supervision of JD. The statistical analysis was performed by JD and both authors contributed to the writing of the manuscript.

## Funding

This research has been partially funded by grants PSI2015-65689-P and SEV-2015-0490 from the Spanish Government, PI2015-1-27 from the Basque Government, AThEME-613465 from the European Union, ERC-AdG-295362 grant from the European Research Council, a grant from the Fundación BBVA and BEX 1692-13-5 grant from the CAPES Foundation.

### Conflict of interest statement

The authors declare that the research was conducted in the absence of any commercial or financial relationships that could be construed as a potential conflict of interest.
