# Supplementary material for: Not Everybody Sees the Ness in the Darkness: Individual Differences in Masked Suffix Priming
Source: Front Psychol. 2016 Oct 14;7:1585. doi: 10.3389/fpsyg.2016.01585 (PMC5063847; doi:10.3389/fpsyg.2016.01585)
Supplement: Supplementary file 1 [file DataSheet1.docx]

**Appendix**

List of item pairs used in the suffix priming lexical decision task. The words presented in uppercase correspond to the targets, and the words presented in lowercase correspond to the primes.

*monedero-TAPICERO; humorista-RACISTA; barrigón-PAREDÓN; blancura-AMARGURA; diccionario-FUNCIONARIO; liderato-CELIBATO; anunciante-TRAFICANTE; algodonoso-GELATINOSO; probador-GOLEADOR; pescador-TIRADOR; relojero-PANADERO; triunfador-NADADOR; timbrazo-LATIGAZO; tontería-RELOJERÍA; tabernero-MONTAÑERO; maletero-BARBERO; comunismo-MONTAÑISMO; domador-GANADOR; definitorio-DORMITORIO; penoso-PEGAJOSO; armador-MIRADOR; espinoso-TEMEROSO; sancionador-COLABORADOR; bajura-RICURA; armazón-CHILLÓN; tacañería-ALBAÑILERÍA; terrorista-FUTBOLISTA; guerrero-FRUTERO; analizador-MADRUGADOR; cartero-COCINERO; consejero-PETROLERO; amador-ORADOR; feminismo-HUMANISMO; ciclismo-MACHISMO; humanista-BAJISTA; medallón-APAGÓN; escudero-TESORERO; veraneante-VOTANTE; lanzador-ANIMADOR; costero-LIBRERO; comprador-NARRADOR; mujeriego-VERANIEGO; modista-PESIMISTA; ilusionismo-DETERMINISMO; andante-INSULTANTE; panero-ROPERO; cargamento-FUNDAMENTO; clasista-BROMISTA; espumoso-VERDOSO; segundero-JARDINERO; ventilador-CONSUMIDOR; emprendedor-ENTENDEDOR; cabezón-ESTIRÓN; destilador-AGITADOR; centrista-FEMINISTA; visitante-INTERROGANTE; dibujante-TOLERANTE; cristalería-PORTERÍA; excitante-DONANTE; machista-ELITISTA; aplastante-PRACTICANTE; cabecero-PORTERO; pajarería-TESORERÍA; debutante-VOLANTE; torero-LIMONERO; racismo-ESPEJISMO; modernismo-EXTREMISMO; salvador-VENDEDOR; gritón-RESULTÓN; pensador-ACOMODADOR; negociador-CREADOR; conocedor-EXPORTADOR; voladura-PICADURA; tapicería-PANADERÍA; destilería-JARDINERÍA; secretario-MILLONARIO; detonante-REPELENTE; tendero-GRANERO; patrullero-PRISIONERO; competidor-REMOLCADOR; esbeltez-SOLIDEZ; ocupante-CANTANTE; colador-ESQUIADOR; dictador-FUNDADOR; navideño-HOGAREÑO; velador-CALCULADOR; pesimismo-SEXISMO; vejestorio-ACLARATORIO; sexista-MOTORISTA; barbudo-CORNUDO; martillazo-TELEFONAZO; trabajador-DETONADOR; imitador-BOXEADOR; organizador-ESTIMULADOR; contador-LUCHADOR; zapatazo-PICOTAZO; obligatorio-LABORATORIO; pasante-CALMANTE; determinante-NAVEGANTE; ignorante-VIGILANTE; absolutista-PROGRESISTA; deportista-MODERNISTA; comedero-MATADERO; preparador-ENCENDEDOR; aspirador-DISEÑADOR; paracaidismo-OPTIMISMO; legionario-ORIGINARIO; negociante-CAMBIANTE; apuntador-MERECEDOR; corredor-CAZADOR; operatorio-ESCRITORIO; consejería-CERVECERÍA; secundario-BANCARIO; organista-VELOCISTA; arenoso-CARNOSO; picajoso-VISTOSO; pensionista-TELEFONISTA; tazón-TIRÓN; triunfante-INTEGRANTE; mechero-PISTOLERO; barbería-LIBRERÍA; tartamudez-EXQUISITEZ; temerario-SOLIDARIO; respiratorio-OBSERVATORIO; papelería-ZAPATERÍA; soldadura-QUEMADURA; borroso-CALUROSO; saltador-OBSERVADOR; fumador-SIMULADOR; marcador-SOLDADOR; afilador-EDUCADOR; artista-BAÑISTA; curandero-AZUCARERO; soñador-SEGADOR; pacifismo-CENTRISMO; quemazón-GIGANTÓN; hablante-VIAJANTE; progresismo-ALCOHOLISMO; contestador-TORTURADOR; comedor-CARGADOR; madrugón-ACELERÓN; lavadero-FUMADERO; gigantismo-POSITIVISMO; mediador-SERVIDOR; directorio-PURGATORIO; ayudante-PASEANTE; contenedor-EXPLORADOR; idiotez-CALIDEZ; entrenador-CONGELADOR; coladero-APEADERO; habitante-OXIDANTE; montañoso-ESPONJOSO; churrero-LETRERO; delgadez-SENSATEZ; rotatorio-VELATORIO; igualdad-GRAVEDAD; carcelero-HECHICERO; computador-GOBERNADOR; mostrador-PECADOR; cuidador-SURTIDOR; cursilería-FRUTERÍA; cafetería-GUARDERÍA; bebedor-GENERADOR; rapidez-ESCASEZ; tendedero-VERTEDERO; minería-PIRATERÍA; misionero-COCOTERO; papelón-RICACHÓN; sujetador-RECIBIDOR; medidor-VENCEDOR; escudería-HECHICERÍA; juramento-PEGAMENTO; admirador-ESCALADOR; poseedor-SEGUIDOR; palidez-RIGIDEZ; borrón-LLORÓN; tontorrón-CUARENTÓN; imaginería-CUBERTERÍA; ilusionista-CONGRESISTA; amplificador-PATROCINADOR; cenicero-MINERO; colorista-PACIFISTA; picadero-FREGADERO; fulminante-DIRIGENTE; mandatario-VECINDARIO; pastelero-ENFERMERO; protestante-CONCURSANTE; sillón-GRUÑÓN; volador-CRIADOR; justificante-PARTICIPANTE; archivador-ACUSADOR; gobernante-ESTUDIANTE; visitador-ACELERADOR; extremista-CARTERISTA; nitidez-ROBUSTEZ; amortiguador-SECUESTRADOR; caminante-HUMILLANTE; giratorio-SANATORIO; realizador-FIJADOR; investigador-CONQUISTADOR; limpiador-VIVIDOR; perdedor-PROVEEDOR; militarismo-ABSOLUTISMO; hombretón-CONTESTÓN; izquierdista-PARACAIDISTA; emisario-IMAGINARIO; ligadura-ARMADURA; vocabulario-SEXAGENARIO; pianista-CICLISTA; adaptador-COORDINADOR; levantador-PROGRAMADOR; adiestrador-ESPECULADOR; soplón-BLUSÓN; pecaminoso-VOLUMINOSO; optimista-COMUNISTA; picante-DELINEANTE; ajedrecista-GUITARRISTA; enfermería-PASTELERÍA; regulador-DECORADOR; jugador-INDICADOR; estimulante-COMERCIANTE; golfista-EBANISTA; moralista-SIMPLISTA; hervidero-CRIADERO; cargante-MILITANTE; herrero-BASURERO; flotador-HABLADOR; sureño-ISLEÑO; pajarero-MAÑANERO; portón-MECHÓN; sesentón-DORMILÓN; acogedor-BORRADOR; refranero-CAMIONERO; purgante-EMIGRANTE; operador-COBRADOR; frenazo-PORTAZO; brumoso-CANOSO; apendicitis-ENCEFALITIS; moderador-ROTULADOR; principiante-SIMPATIZANTE; barrigudo-CABELLUDO; pluralismo-TERRORISMO; amatorio-ORATORIO; criatura-FRESCURA; ligamento-ARMAMENTO; vaporoso-ACEITOSO; añadidura-DICTADURA; solicitante-CHISPEANTE; herrería-CACERÍA; brasero-ZAPATERO.*
